# The potential for arms race and Red Queen coevolution in a protist host–parasite system

**DOI:** 10.1002/ece3.1314

**Published:** 2014-12-02

**Authors:** Lars Råberg, Elisabet Alacid, Esther Garces, Rosa Figueroa

**Affiliations:** 1 Department of Biology, Lund UniversityEcology Building, SE-22362, Lund, Sweden; 2 Departament de Biologia Marina i Oceanografia, Institut de Ciències del Mar, CSICPg. Marítim de la Barceloneta, 37-49, E08003, Barcelona, Spain; 3 Centro Oceanográfico de Vigo, IEO (Instituto Español de Oceanografía)Subida a Radio Faro 50, 36390, Vigo, Spain

**Keywords:** *Alexandrium minutum*, dinoflagellate, fluctuating selection, frequency-dependent selection, *Parvilucifera sinerae*

## Abstract

The dynamics and consequences of host–parasite coevolution depend on the nature of host genotype-by-parasite genotype interactions (G × G) for host and parasite fitness. G × G with crossing reaction norms can yield cyclic dynamics of allele frequencies (“Red Queen” dynamics) while G × G where the variance among host genotypes differs between parasite genotypes results in selective sweeps (“arms race” dynamics). Here, we investigate the relative potential for arms race and Red Queen coevolution in a protist host–parasite system, the dinoflagellate *Alexandrium minutum* and its parasite *Parvilucifera sinerae*. We challenged nine different clones of *A. minutum* with 10 clones of *P. sinerae* in a fully factorial design and measured infection success and host and parasite fitness. Each host genotype was successfully infected by four to ten of the parasite genotypes. There were strong G × Gs for infection success, as well as both host and parasite fitness. About three quarters of the G × G variance components for host and parasite fitness were due to crossing reaction norms. There were no general costs of resistance or infectivity. We conclude that there is high potential for Red Queen dynamics in this host–parasite system.

## Introduction

Hosts and parasites are by definition in conflict with each other. Parasites can therefore be expected to impose selection for host resistance, while hosts should select for enhanced infectivity of parasites. This reciprocal selection can result in coevolution, with continuous changes of both host resistance and parasite infectivity. At the genetic level, coevolution can take two fundamentally different forms: successive fixation of advantageous mutations (selective sweeps), or cyclic dynamics of allele frequencies. Following Woolhouse et al. (2002), we refer to these two types of dynamics as “arms race” and “Red Queen” coevolution, respectively (Red Queen dynamics are also known as “fluctuating selection dynamics”; e.g., Hall et al. 2011). Importantly, the two types of coevolution have radically different consequences; arms races lead to rapid evolution of the genes involved but generally low levels of standing genetic variation, whereas Red Queen dynamics result in balanced polymorphisms with deep coalescence times (Bergelson et al. 2001). Moreover, only Red Queen dynamics favor recombination and sexual reproduction (Parker 1994; Agrawal and Lively 2002; Morran et al. 2011). It would therefore be of great interest to understand the relative importance of these two modes of coevolution in nature.

The most direct way to distinguish arms race and Red Queen dynamics is to test the predictions of the two scenarios through “time shift experiments”, where hosts are challenged with parasites from past, contemporary and future generations (or vice versa; Gaba and Ebert 2009). A number of such experiments have been performed with bacteria (primarily *Pseudomonas fluorescens*) and viral parasites (phage). In bacteria-phage systems, coevolution experiments typically result in arms race dynamics (Brockhurst et al. 2007), although this may be a consequence of experimental design (relatively short experiments, no standing genetic variation at start, and nutrient rich medium). Indeed, an experiment with *Pseudomonas* and phage (Hall et al. 2011) showed that coevolution initially followed the arms race scenario, but eventually turned into Red Queen dynamics, presumably as a result of increasing costs of host resistance and parasite infectivity. Moreover, an experiment in soil, where costs of resistance are more pronounced than in traditional lab medium, resulted in fluctuating dynamics (Gómez and Buckling 2011; see also Koskella 2013). Time shift experiments have also been performed with *Daphnia* and a bacterial pathogen, a snail-trematode system, and a plant-fungal rust system (Decaestecker et al. 2007; Koskella and Lively 2009; Thrall et al. 2012). In these cases, patterns are consistent with fluctuating dynamics. Taken together, the experiments performed to date suggest Red Queen dynamics are the dominant mode of coevolution in nature (at least over ecological time scales), but more studies from other systems are clearly desirable to confirm this impression. Unfortunately, time shift experiments are often logistically demanding. Moreover, the number of systems where it is possible to retrieve ancient hosts and parasites from natural populations (like in *Daphnia;* Decaestecker et al. 2007) is limited.

**Figure 1:**
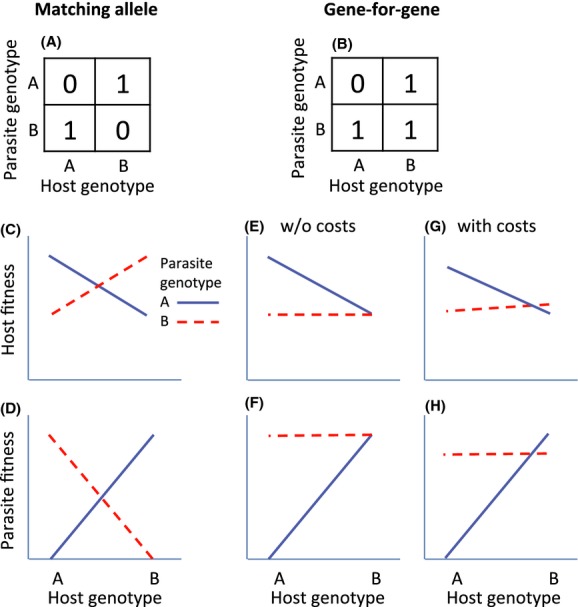
Infection success and host and parasite fitness under matching allele and gene-for-gene G × G. (A) Infection success under matching allele scenario. (B) Infection success under gene-for-gene scenario. (C) Host fitness under matching allele scenario. (D) Parasite fitness under matching allele scenario. (E) Host fitness under gene-for-gene scenario without costs of resistance and infectivity. (F) Parasite fitness under gene-for-gene scenario without costs. (G) Host fitness under gene-for-gene scenario where host A pays a cost of resistance and parasite B pays a cost of infectivity. (H) Parasite fitness under gene-for-gene scenario where host A pays a cost of resistance and parasite B pays a cost of infectivity. The figures were drawn using the equations in (Agrawal and Lively 2002), assuming a virulence of 0.5 (i.e., the reduction of fitness of infected hosts), a cost of infectivity of 0.1, and a cost of resistance of 0.05.

An alternative approach to gain insight into the relative importance of arms race and Red Queen dynamics is to test the assumptions of the respective scenario using samples of contemporary hosts and parasites. Theoretical models of host–parasite coevolution are based on the assumption that there is specificity between host and parasite genotypes, that is, a host genotype-by-parasite genotype interaction, for infection success [henceforth G × G; Woolhouse et al. (2002)]. G × Gs can take two fundamentally different forms: “gene-for-gene” (GFG) and “matching allele” (MA) interactions (Frank 1993; Agrawal and Lively 2002; Kover 2006). In the matching allele scenario, each parasite genotype can only infect one (or a subset of) host genotypes (Fig. 1A). In contrast, in the classical GFG scenario, there are universal infectivity and susceptibility genotypes, so that one parasite genotype can infect all hosts while others can only infect a few host genotypes (Fig. 1B).

The type of coevolutionary dynamic expected in a given host–parasite system depends on how the G × G for infection success translates into G × Gs for host and parasite fitness. This in turn depends on the nature of costs of host resistance and parasite infectivity. In MA, the cost of host resistance to one parasite genotype is expressed as increased susceptibility to other genotypes, while the cost of parasite infectivity to one host genotype is lack of infectivity to another. Assuming that infection reduces host fitness, this kind of specificity for infection success should result in that the ranking of host genotypes with respect to fitness differs between parasite genotypes (Fig. 1C). Similarly, assuming that inability to infect a host reduces parasite fitness, MA G × G for infectivity should result in that the ranking of parasite genotype with respect to fitness differs between host genotypes (Fig. 1D). Following Barrett et al. (2005), we refer to this type of G × G as “inconsistency” G × G. In contrast, in GFG, there may or may not be general costs of resistance and infectivity. We here follow the definitions of such costs given by Agrawal and Lively (2002). Accordingly, costs of resistance should be expressed as reduced fitness in the absence of parasites, while costs of infectivity mean that parasites with a broad host range should have lower transmission from infected hosts than parasites with a more narrow host range (following the “jack of all trades, master of none” logic). The translation of GFG G × G for infectivity into host and parasite fitness depends on the presence of such costs. In the absence of costs, the GFG scenario results in that the variance in host fitness across parasite genotypes differs between host genotypes, and vice versa (Fig. 1E and F), that is, a “responsiveness” G × G (Barrett et al. 2005). With costs of resistance and infectivity, GFG translates into a G × G for host and parasite fitness that has an element of both “responsiveness” and “inconsistency” (i.e., there are differences in both variance and ranking; Fig. 1G and H).

G × Gs for host and parasite fitness that are purely a result of differences in variance (“responsiveness” G × G) lead to arms race coevolution, but as soon as there are some differences in ranking (“inconsistency” G × G), there can be cyclic Red Queen dynamics (Agrawal and Lively 2002). The more of the G × G that is due to differences in ranking (as the system moves from GFG to MA, or there are higher costs of infectivity and resistance), the more pronounced will the Red Queen dynamics be (higher amplitude, shorter period, and more even allele frequencies; Agrawal and Lively 2002). By dissecting the relative contribution of differences in variance and differences in ranking to G × Gs for host and parasite fitness, it should therefore be possible to predict the nature of the coevolutionary dynamics.

G × Gs for infection success or quantitative measures of host and parasite fitness-related traits have been demonstrated in a number of different host–parasite systems (Carius et al. 2001; Schulenburg and Ewbank 2004; Lambrechts et al. 2005; Rauch et al. 2006; Salvaudon et al. 2007; De Roode and Altizer 2010; Carpenter et al. 2012; Luijckx et al. 2013), but the relative importance of “inconsistency” and variation in “responsiveness” to these G × Gs has rarely been investigated (but see De Roode and Altizer 2010; Luijckx et al. 2013). Hence, little is as yet known about the mode of coevolution in these systems.

Here, we investigate the relative potential for arms race and Red Queen dynamics in a protist host–parasite system, the dinoflagellate host *Alexandrium minutum* and the parasite *Parvilucifera sinerae*. To this end, we first tested for G × Gs for infection success and host and parasite fitness, and then dissected the observed G × Gs for host and parasite fitness into “inconsistency” and “responsiveness”. We also tested whether there were costs of resistance and infectivity.

## Materials and Methods

### Study system

The study system is formed by the dinoflagellate *A. minutum* (Halim 1960) and the Perkinsozoa parasite *P. sinerae* (Figueroa and Garcés 2008). *Alexandrium minutum* is a cosmopolitan toxic microalgae which blooms worldwide, with blooms being especially recurrent and intense at some Mediterranean sites (Bravo et al. 2008; Garcés and Camp 2012). *Parvilucifera sinerae* was first described in Arenys de Mar (NW Mediterranean sea, Spain) during an *A. minutum* bloom (Figueroa et al. 2008). The genus *Parvilucifera* is a generalist parasite on dinoflagellates (e.g., Garcés et al. 2012).

The complete life cycle of *A. minutum* is complex and involves shifts between haploidy and diploidy, representing planktonic and benthic forms, respectively. Haploid planktonic *A. minutum* cells generally divide mitotically (something which allows for maintenance of clonal cultures in the lab). However, haploid stages may act as gametes which fuse to form zygotes and then either divide (presumably by meiosis) or become resting cysts which survive in the sediments during long periods of time if necessary. In *A. minutum*, two different and compatible mating types (clones) need to be mixed to have sexual reproduction and produce resting cysts (Figueroa et al. 2008).

**Figure 2:**
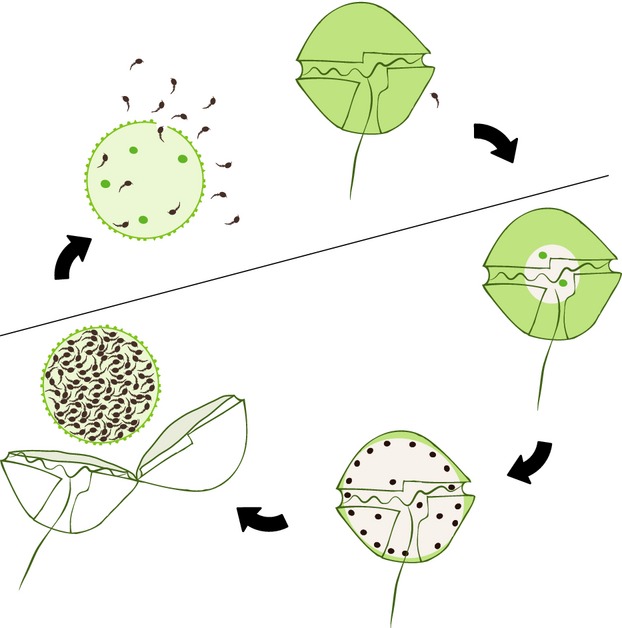
Schematic infection cycle of *Alexandrium minutum* by *Parvilucifera sinerae*. A zoospore of the parasite enters into the host, replicates, and forms a sporangium filled with dormant zoospores. To complete the cycle, zoospores are activated and burst the host cell to infect new host cells. In this study, the term sporangium refers all stages of infection as indicated below the line.

The infection cycle of the parasite proceeds as shown in Figure 2. Briefly, a flagellate zoospore penetrates the host cell, destroys its content, and progressively forms a spherical sporangium of the same size as the host, filling up the sporangium with about 250 dormant zoospores. The sporangium remains dormant until chemical signals, such as the presence of a sufficient density of host cells, activates zoospores and they leave the sporangium to find another host for infection. The duration of the whole maturation of sporangium in *A. minutum* is about 4 days (Figueroa et al. 2008, Garcés et al. 2012).

In the present experiment, we used 9 clonal strains of the host (each clone established from a single vegetative cell) and 10 clonal strains of the parasite (each clone established from a single sporangium) isolated from the northwestern Mediterranean Sea. Host cultures belong to the Instituto Español de Oceanografía (Vigo, Spain) culture collection. To ensure clonality of both host and parasite, they were cloned 3 months before the experiment (January 2012). Details of host and parasite clones are given in Tables S1 and S2, respectively.

### Experiment

Host cultures were grown at 15°C with an irradiance of approximately 50 *μ*mol photons m^−2·^s^−1^ and a photoperiod of 12:12 h L:D (light:dark). Culture stocks were maintained in Iwaki 50 mL flasks filled using L1 medium (Guillard and Hargraves 1993) without added silica. To generate inocula, parasite clones were amplified on the clonal *A. minutum* strain VGO650, isolated in Brittany (France), in 2003. The medium was prepared using Baltic seawater adjusted to a salinity of 28 psu. Three hundred and sixty flasks (9 × 10 matrix of all possible host and parasite combinations in four replicates) at an initial density of 3000 cells mL^−1^ (20 mL total volume) were infected with five parasite sporangia (zoospore: host ratio of 1:60). Our choice of dose was based on the following reasoning: The dose should be as low as possible to mimic the initial phase of an epidemic. However, if the dose is too low, the variability between replicates increases, because the probability of failing either with the inoculation or in selecting viable sporangia increases. Based on pilot experiments, we determined that five sporangia were enough to minimize the variation among replicates. Uninfected cultures (36 flasks, four replicates of each host clone) were used as controls. From each flask, 1 mL was sampled on every second day from day 2 to 16 days (except day 12). The experiment was performed in March 2013 at the Department of Biology, Lund University. Samples were fixed with formalin (1%) and placed in Sedgewick-Rafter chambers (SPI, West Chester, PA) for cell and sporangia enumeration. At least 300 cells were counted. Mature sporangia were identified following Figueroa et al. (2008).

### Analyses

We measured the outcome of infection in three ways: infection success and host and parasite fitness. Infection success measures whether the parasite was able to infect and replicate or not and was scored as 1 or 0 for each flask depending on whether sporangia were observed on at least 1 day during day 2–16 or not. Infection success was analyzed by means of a generalized linear model with binomial distribution, with host clone, parasite clone, and their interaction as random effects, using the glimmix procedure in SAS 9.3 (SAS Inc., Cary, NC). Parameters were estimated by Laplace approximation and *P*-values determined by likelihood ratio tests, as recommended by Bolker et al. (2009). The test statistic for likelihood ratio tests was calculated as *G*^2^ = −2(log likelihood reduced model-log likelihood full model) and compared against a *χ*^2^ distribution with 1 df (Quinn and Keough 2002). To test for effects of geographic distance or temporal difference between host and parasite isolates on infection success, we performed a generalized linear model (proc glimmix; binomial distribution) with infection success (0 or 1) against geographic distance and temporal difference (year parasite isolated-year host isolated), with intercept and slope as random effects and parasite as “subject”. The response variable was modeled as “events/trials”, that is, number of flasks where infection was successful out of the four flasks with each host–parasite combination.

Host and parasite fitness were measured as net growth/mortality rates. We chose these measures because they should best reflect fitness in expanding/contracting populations. Other measures of host and parasite fitness, for example, total abundance of hosts/parasites during the experiment, or maximum proportion of infected hosts, yielded the same conclusions. Growth/mortality rates of hosts and parasites were calculated as *K*_10_ = log (*N*_1_/*N*_0_)/(*t*_1_ − *t*_0_), where *N* is the number of cells and *t* is time (in days; Guillard 1973). We tested for differences in host fitness between inoculated flasks (regardless of parasite genotype) and un-inoculated controls by means of a general linear mixed model with experiment (inoculated or not) as fixed factor and host genotype as random effect. We tested for G × G for host and parasite fitness by means of random effects models, with host clone, parasite clone, and their interaction as random effects. These analyses were performed with proc mixed in SAS 9.3. *P*-values for random effects were determined by *F* tests (method = type 3), as recommended when there are relatively few levels of the random effects (Littell et al. 2006)(Wald tests yielded similar conclusions, in particular as regards the significance of host genotype-by-parasite genotype interaction terms). Diagnostic plots were checked to ensure that residuals were normally distributed.

To dissect the relative contribution of differences in variance (“responsiveness”) and differences in ranking (“inconsistency”) to G × Gs for host and parasite fitness, we used the method of Cockerham (1963; see also Fry et al. 1996). Briefly, the variance due to G × G can be calculated as


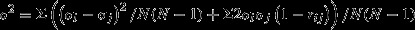
(1)

where *σ*_*i*_ is the square root of the variance component among host strains in parasite *i, r*_*ij*_ is the correlation between host strains in parasite *i* and *j,* and *N* is the number of parasite genotypes. The first component in Eqn 1 represents G × G due to differences in variance (“responsiveness”), while the second component represents G × G as a result of differences ranking (“inconsistency”). Variance components were estimated with proc varcomp in SAS 9.3.

To test for effects of geographic distance or temporal difference between host and parasite isolates on host and parasite fitness, we performed general linear mixed models (proc mixed) with host or parasite fitness against geographic distance and temporal difference, with intercept and slope as random effects and parasite as “subject”.

To test for costs of resistance and infectivity, we followed the definitions of resistance and infectivity given by Agrawal and Lively (2002; note that they follow the plant literature and use the term virulence instead of infectivity). Host resistance was calculated by first taking the average proportion of flasks of each host–parasite combination where infection was successful and then taking the average of that for each host genotype (note that a higher value means lower resistance). Parasite infectivity (essentially host range) was calculated by first taking the average proportion of flasks of each host–parasite combination where infection was successful and then taking the average of that for each parasite genotype.

## Results

### Basic population dynamics of host

**Figure 3:**
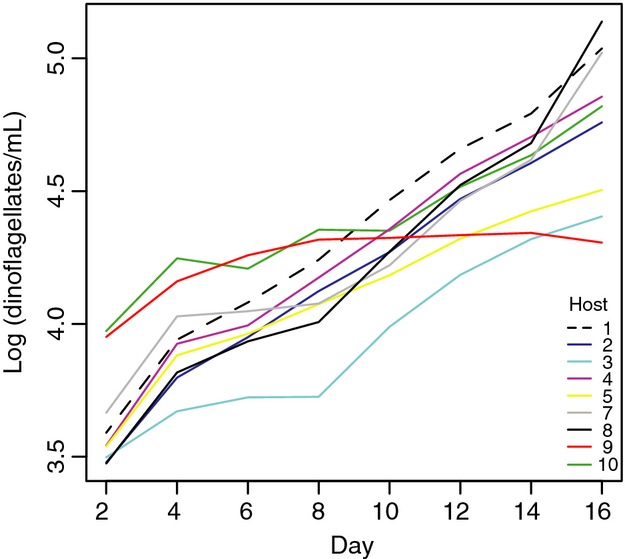
Cell abundance of uninfected controls of each strain (each line represents the average of four flasks).

The population dynamics of uninfected controls are shown in Figure 3. All genotypes were in exponential growth throughout the experiment, with exception of host 9 which reached carrying capacity already on day 8–10. Host genotype explained 97.4% of the variation in growth rate from day 2–16 *F*(8, 27) = 149.3, *P* < 0.0001.

### Infection success

**Figure 4:**
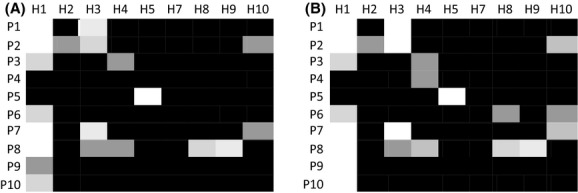
Proportion successful infections in different host–parasite combinations, from 0% (white) to 100% (black). In (A) infection success is scored as 1 or 0 depending on whether sporangia where observed at any time during day 2–16. In (B) infection success is scored as 1 or 0 depending on whether infection established (sporangia present on day 14 and/or 16) or not.

Of the 90 host–parasite combinations, 70 were completely compatible (infection occurred in 4/4 flasks), five were completely incompatible (infection occurred in 0/4), and 15 were partly compatible (infection occurred in 1/4–3/4; Fig. 4A). The host × parasite interaction was highly significant (likelihood ratio test: *G*^2^ = 32.6, df = 1, *P* < 0.001).

In most flasks where sporangia were observed, the parasite established and the host population declined. However, in some flasks, a few sporangia were observed early during the infection, but the parasite did not establish an infection (no sporangia observed at the end of the experiment) and the host continued to grow exponentially. If infection success is scored as 0 or 1 depending on whether infection established (sporangia present on day 14 and/or 16) or not, instead of whether sporangia were observed at all, the host × parasite interaction is even stronger (Fig. 4B; likelihood ratio test: *G*^2^ = 52.3, df = 1, *P* < 0.001).

Infection success was dependent on the temporal difference between parasite and host isolates *F*(1, 9) = 36.9, *P* = 0.0002, so that infection success decreased with temporal difference. Infection success was not dependent on the geographic distance between host and parasite isolates *F*(1, 9) = 0.09, *P* = 0.76. The effect of temporal difference on infection success was driven by host isolate 1 (which was isolated earlier than the others; see Table S1). When this isolate was removed, there was no effect of temporal difference on infection success (*P* = 0.15). The host × parasite interaction for infection success was not dependent on the temporal difference between isolates; it was highly significant also when host isolate 1 was removed from the analysis (*G*^2^ = 38.5, df = 1, *P* < 0.001).

### Host fitness

**Figure 5:**
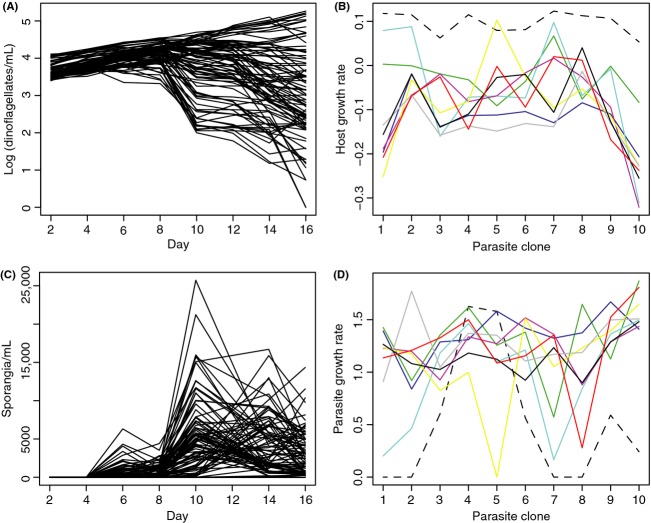
(A) Population dynamics of hosts (infected + uninfected) in inoculated flasks. Each line represents one host–parasite combination (average of four replicate flasks). (B) Fitness of the different host genotypes (measured as change in population density during day 4–16) when inoculated with each of the ten parasite clones. Each line represents one host clone (average of four flasks for each host–parasite combination). (C) Population dynamics of the parasite. Each line represents one host–parasite combination (average of four replicate flasks). We show parasite dynamics on raw scale (rather than log scale as for the host) because this makes the fluctuating dynamics more clear. (D) Fitness of the different parasite genotypes (measured as maximum growth rate during a 2-day period during day 2–16) when inoculated in cultures of each of the nine host genotypes. Each line represents one host genotype (average of four flasks for each host–parasite combination).

Host population densities over time for each host–parasite combination are presented in Figure 5A. Population densities increased in all flasks from day 2 to 4, but from day 4 onwards growth trajectories varied from exponential growth (in resistant hosts) to more or less exponential mortality (in susceptible hosts). As a measure of host fitness during infection, we used the change in population density from day 4 to 16.

**Figure 6:**
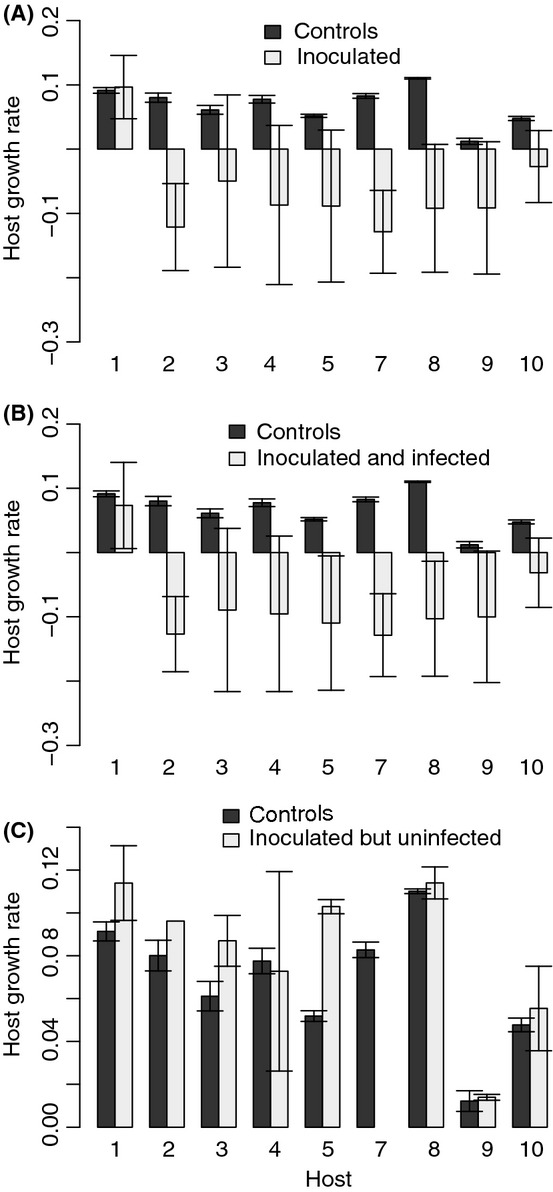
Growth rates of different host genotypes. (A) Inoculated versus un-inoculated controls. (B) Inoculated where infection was successful versus controls. (C) Inoculated where infection was unsuccessful versus controls.

We first tested for general fitness effects of the parasite by comparing host growth in inoculated flasks (irrespective of parasite genotype) and un-inoculated controls. There was a significant difference between inoculated flasks and un-inoculated controls *F*(1, 8) = 31.81, *P* < 0.0005. However, there was also a significant interaction between host and inoculation *F*(8, 378) = 2.17, *P* = 0.029, indicating that the effect of the parasite varied between host genotypes. Separate analyses for each host genotype showed that six of the host genotypes had lower growth rate in inoculated flasks than un-inoculated controls, while there was no statistically significant difference between treatments for three host genotypes (Fig. 6A). To dissect the effects of infection on host fitness further, we divided inoculated flasks into two categories depending on whether infection was successful or not (i.e., if sporangia were observed or not). A comparison of un-inoculated controls and inoculated where infection was successful showed, not surprisingly, that the parasite had an even stronger effect on host fitness in this subset of the data *F*(1, 8) = 47.36, *P* < 0.0001, but that there was still a host × inoculation interaction *F*(8, 332) = 1.98, *P* = 0.048. Separate analyses for each host showed that in flasks where infection was successful, the parasite had a negative effect on host growth in all host genotypes except host 1 (Fig. 6B). A comparison of un-inoculated controls and inoculated flasks where infection was not successful showed, somewhat surprisingly, that the growth rate was significantly higher in inoculated flasks *F*(1, 7) = 6.66, *P* = 0.036 (host genotype 7 was excluded from this analyses because infection was successful in all inoculated flasks). Again, there was also a significant interaction between host and inoculation *F*(7, 62) = 3.19, *P* = 0.006. Separate analyses for each host genotype showed that host 1, 3, and 5 had higher growth rate in inoculated flasks where infection was not successful than in un-inoculated controls, while there was no difference for the other six host genotypes (Fig. 6C).

To test for G × G for host fitness, we performed an analyses across all inoculated flasks (rather than just flasks where infection was successful, as in for example, De Roode and Altizer (2010)), because we wanted to estimate the fitness of host and parasite in a particular combination, regardless of whether infection was successful or not (because these are the parameters in mathematical models of coevolution; cf Agrawal and Lively 2002). In inoculated flasks, host fitness was influenced by host genotype, parasite genotype, and the host genotype × parasite genotype interaction (Fig. 5B, variance estimates: host = 30.1%, *F*(8, 72) = 11.85, *P* < 0.0001; parasite = 20.5%, *F*(9, 72) = 7.46, *P* < 0.0001; host × parasite = 21.9%, *F*(72, 270) = 4.28, *P* < 0.0001). 78.7% of the interaction term was a result of “inconsistency” and 21.3% variation in “responsiveness”.

Host fitness was dependent on the temporal difference between parasite and host isolates *F*(1, 9) = 52.5, *P* < 0.0001, so that host fitness increased with temporal difference. There was also an effect of geographic distance between host and parasite isolates *F*(1, 9) = 11.4, *P* = 0.0083. As for infection success, these effects were driven by host isolate 1. When this isolate was removed, there was no effect of temporal difference on host fitness (*P* = 0.39) and no effect of geographic distance (*P* = 0.28). The host × parasite interaction for host fitness was not dependent on the temporal difference or geographic distance between isolates; it was highly significant also when host isolate 1 was removed from the analysis *F*(63, 240) = 4.32, *P* < 0.001.

### Parasite fitness

Parasite population dynamics over time for each host–parasite combination are presented in Figure 5C. In a large proportion of the flasks, there was a first peak on day 6 followed by a second higher peak on day 10, where after the parasite population declined as the host population crashed. In others, the parasite grew more slowly and did not show a first peak until day 10 or 14. Because of the nonlinear dynamics of the parasite in many flasks, growth rates over a longer time period could not be easily calculated (in contrast to the host, which typically showed a steady increase/decline from day 4 to 16; see above). Moreover, because the growth rate of the parasite is subject to negative feedback (a rapidly growing parasite will reduce the density of susceptible hosts and thereby its own growth rate), calculating parasite growth rates over a longer time period would not reflect its ability to spread in the host population. As a measure of parasite fitness, we therefore chose to use the maximum growth rate during a 2-day period from day 2 to 16.

Like the analysis of G × G for host fitness above, the test for G × G for parasite fitness was performed across all inoculated flasks, regardless of whether infection was successful or not. Parasite fitness was influenced by host genotype, parasite genotype, and the host × parasite interaction (Fig. 5D; variance estimates: host = 17.6%, *F*(8, 72) = 5.36, *P* < 0.0001; parasite = 7.2%, *F*(9, 72) = 2.62, *P* = 0.011; host × parasite = 28.9%, *F*(72, 270) = 3.50, *P* < 0.0001). 75.5% of the interaction term was a result of “inconsistency” and 24.5% variation in “responsiveness”.

Parasite fitness was dependent on the temporal difference between parasite and host isolates *F*(1, 9) = 16.5, *P* = 0.0029, so that parasite fitness decreased with temporal difference. There was no effect of geographic distance between host and parasite isolates *F*(1, 9) = 0.01, *P* = 0.91. As for infection success, the effect of temporal difference was driven by host isolate 1. When this isolate was removed, there was no effect of temporal difference on parasite fitness (*P* = 0.12) and no effect of geographic distance (*P* = 0.12). The host × parasite interaction for parasite fitness was not dependent on the temporal difference or geographic distance between isolates; it was highly significant also when host isolate 1 was removed from the analysis *F*(63, 240) = 2.83, *P* < 0.001.

### Cost of resistance and infectivity

To test for costs of resistance, we tested for a correlation between host fitness (growth rate) in uninfected controls and resistance, using host genotype as the unit of analysis. There was no correlation between growth rate and resistance (*r*_S_ = 0.17, *P* = 0.66).

To test for costs of infectivity, we tested for a negative correlation between parasite fitness in flasks where infection was successful and infectivity (host range), using parasite genotype as the unit of analysis. There was no correlation between parasite fitness and host range (*r*_S_ = 0.46, *P* = 0.18).

## Discussion

We found a strong G × G for infection success, which translated into G × Gs for both host and parasite fitness. The G × Gs for fitness were mainly a result of “inconsistency”, that is, the reaction norms of different genotypes crossed each other. Based on this, we conclude that there is potential for coevolution between *A. minutum* and *P. sinerae*, primarily Red Queen dynamics. There was no correlation between host resistance and growth rate in the absence of parasites, and no correlation between parasite infectivity and host range, hence no general costs of resistance and infectivity. Instead, the G × Gs seem to primarily be a result of a matching allele pattern where high host resistance (or low parasite infectivity) against some parasites (or hosts) comes at a cost of low resistance (infectivity) against others.

An important question is to what extent are the patterns observed in the lab generalizable to the natural environment? We address four potential caveats of our setup. First, there is no turbulence in the flasks (in contrast to the sea) and the nutrient level in the medium we used is considerably higher than in seawater (we used traditional medium because it is difficult to grow dinoflagellates in seawater under laboratory conditions). Higher nutrient levels mean the host can reach higher population densities, which should enhance the parasite's encounter rate with susceptible hosts. Similarly, lack of turbulence makes it easier for the parasite to attach to host cells (Llaveria et al. 2010). Thus, the infection success in our experiment is likely higher than in nature. However, we cannot see a reason why the G × Gs for infection success should be artefacts of unnaturally high nutrient levels or lack of turbulence.

Second, the nutrient level can also affect the expression of costs of resistance. Life-history trade-offs in general are often more pronounced in harsh environments, and several studies have found that costs of resistance were only present under low nutrient availability (e.g., McKean et al. 2008; Gómez and Buckling 2011). Hence, the absence of costs in our study could be a result of too benign conditions. If costs are present under natural conditions, some of ca 25% “responsiveness” G × G for host and parasite fitness could turn into “inconsistency” G × G. Our setup might therefore overestimate the scope for arms race coevolution.

Third, the host and parasite clones in our experiment originated from a large area of the western Mediterranean Sea and from several different years (see Tables S1 and S2). In contrast, blooms of *A. minutum* and ensuing epidemics of *P. sinerae* are more isolated phenomena both in space and time; dinoflagellate blooms typically occur at harbors and other nutrient rich coastal sites. The wide geographic and temporal origin of isolates (in particular hosts) mean that the G × Gs could potentially be influenced by local adaptation of parasites (or hosts). For instance, the parasite could be expected to be more successful on sympatric than allopatric hosts (cf. Ebert 1994). There were indeed effects of temporal difference and geographic distance, but these were driven by a single host isolate (which was isolated earlier than the others) and did not explain the G × Gs. As the isolates had a wide geographic and temporal origin, one could also expect that the standing genetic variation across our flasks is higher than in a bloom. Yet, microsatellite-based analyses of a closely related species, *Alexandrium fundyense,* showed that genetic diversity in *Alexandrium* blooms can be high, with extensive clonal diversity (Richlen et al. 2012). Hence, the amount of genetic variation in our setup can reflect the situation in natural populations reasonably well.

Given that our setup can be generalized to the natural environment of *A. minutum* and *P. sinerae*, the present study conforms with the general pattern from time shift experiments in that Red Queen dynamics seem to be the most common mode of coevolution (Decaestecker et al. 2007; Koskella and Lively 2009; Gómez and Buckling 2011; Thrall et al. 2012). Hence, coevolution could be an important factor maintaining genetic diversity and favoring recombination and sexual reproduction.

In our analyses, we have followed the scenario outlined by Agrawal and Lively (2002), where infectivity and resistance are qualitative traits that determine whether a host gets infected or not (infection success is either 0 or 1 from both the parasite's and host's perspective) and that this in turn determines the fitness of host and parasite. An alternative scenario is to view infection as a two-step process where the first step reflects whether the parasite can infect or not, and the second to what extent infected hosts can control and eventually clear the infection (Agrawal and Lively 2003). De Roode and Altizer (2010) applied this approach in a study of Monarch butterflies and a protozoan parasite. They found no G × G for infection success, but a strong G × G for infection intensity (number of parasites) in infected hosts, most of which was due to “inconsistency” (69.5%). Hence, the study by De Roode and Altizer indicated that in the Monarch system only genes involved in the second step of infection are potentially involved in coevolution. A similar analysis with the present data set (i.e., a test for G × G for host and parasite fitness among flasks where infection was successful, rather than all inoculated flasks, as in the analyses above), showed there were significant G × Gs also in this subset of the data, primarily in the form of “inconsistency” (64% of G × G for host fitness, and 58% of parasite fitness). Hence, in the case of *A. minutum* and *P. sinerae,* genes involved in both steps of infection (infectivity and subsequent regulation of infection intensities) could potentially be involved in coevolution.

The comparison of host fitness in inoculated flasks and un-inoculated negative controls showed that when the parasite established an infection, it typically had severe effects on host fitness. However, in flasks where the parasite failed to establish, the host often grew faster than in negative controls, suggesting the host can adjust its growth rate in response to perceived threat of parasite infection. Previous studies of dinoflagellates have shown phenotypic plasticity for other life-history traits in response to parasitism (increased sexual reproduction and production of resting cysts; Toth et al. 2004; Figueroa et al. 2010). The ability to increase growth rate may be another way of defending against parasites, that is, by trying to out-grow the parasite. The importance of this kind life-history adjustment as defense against parasitism requires further investigation.

Parasitism, and specifically the genus *Parvilucifera*, has been suggested as a potential biological tool to control harmful algal blooms (Taylor 1968). The high specificity between host and parasite genotypes demonstrated in this study may hamper the application of massive algal infection as a means to control blooms. Moreover, the extensive genetic variation for resistance in the host, in combination with the potential for sexual reproduction, may help the host escape or at least delay the bloom mitigating effect of the parasite.

To conclude, by dissecting the G × Gs for host and parasite fitness, we showed that Red Queen rather than arms race dynamics is likely the dominant outcome of coevolution between *A. minutum* and *P. sinerae*. Unlike time shift experiments, our approach does not yield direct evidence for the nature of coevolutionary dynamics, but rather indicates the potential for Red Queen versus arms races. However, dissection of G × Gs should be applicable to a wider range of host–parasite systems – including vertebrates – than time shift experiments and could therefore be a valuable complement to elucidate the relative importance of Red Queen and arms race dynamics across the tree of life. In the case of *A. minutum* and *P. sinerae,* it would be possible to also perform time shift experiments, and thereby evaluate to what extent the results from the present study actually predicts the nature of coevolutionary dynamics.

## Supporting Information

**Table S1.** Details of host clones. **Table S2.** Details of parasite clones.

## References

[b1] Agrawal A, Lively CM (2002). Infection genetics : gene-for-gene versus matching- alleles models and all points in between. Evol. Ecol. Res.

[b2] Agrawal AF, Lively CM (2003). Modelling infection as a two-step process combining gene-for-gene and matching-allele genetics. Proc. R. Soc. B.

[b3] Barrett RDH, MacLean RC, Bell G (2005). Evolution of pseudomonas experimental fluorescens in simple and complex environments. Am. Nat.

[b4] Bergelson J, Kreitman M, Stahl EA, Tian D (2001). Evolutionary dynamics of plant R-genes. Science.

[b5] Bolker BM, Brooks ME, Clark CJ, Geange SW, Poulsen JR, Stevens MHH (2009). Generalized linear mixed models: a practical guide for ecology and evolution. Trends Ecol. Evol.

[b6] Bravo I, Vila M, Masó M, Figueroa RI, Ramilo I (2008). *Alexandrium catenella* and *Alexandrium minutum* blooms in the Mediterranean Sea: toward the identification of ecological niches. Harmful Algae.

[b7] Brockhurst MA, Morgan AD, Fenton A, Buckling A (2007). Experimental coevolution with bacteria and phage. The Pseudomonas fluorescens–Phi2 model system. Infect. Genet. Evol.

[b8] Carius HJ, Little TJ, Ebert D (2001). Genetic variation in a host-parasite association: potential for coevolution and frequency-dependent selection. Evolution.

[b9] Carpenter JA, Hadfield JD, Bangham J, Jiggins FM (2012). Specific interactions between host and parasite genotypes do not act as a constraint on the evolution of antiviral resistance in Drosophila. Evolution.

[b10] Cockerham CC, Hanson WD, Robertson HF (1963). Estimation of genetic variances. Statistical genetics and plant breeding.

[b11] De Roode JC, Altizer S (2010). Host-parasite genetic interactions and virulence-transmission relationships in natural populations of monarch butterflies. Evolution.

[b12] Decaestecker E, Gaba S, Raeymaekers JAM, Stoks R, Van Kerckhoven L, Ebert D (2007). Host-parasite “Red Queen” dynamics archived in pond sediment. Nature.

[b13] Ebert D (1994). Virulence and local adaptation of a horizontally transmitted parasite. Science.

[b39] Figueroa RI, Garces E, Massana R, Camp J (2008). Description, host-specificity, and strain selectivity of the dinoflagellate parasite Parvilucifera sinerae sp. nov. (Perkinsozoa). Protist.

[b14] Figueroa RI, Garcés E, Camp J (2010). Reproductive plasticity and local adaptation in the host–parasite system formed by the toxic *Alexandrium minutum* and the dinoflagellate parasite *Parvilucifera sinerae*. Harmful Algae.

[b15] Frank SA (1993). Specificity versus detectable polymorphism in host-parasite genetics. Proc. R. Soc. B.

[b16] Fry J, Heinsohn S, Mackay T (1996). The contribution of new mutations to genotype-environment interactions for fitness in Drosophila melanogaster. Evolution.

[b17] Gaba S, Ebert D (2009). Time-shift experiments as a tool to study antagonistic coevolution. Trends Ecol. Evol.

[b18] Garcés E, Stambler N, Camp J (2012). Habitat changes in the Mediterranean Sea and the consequences for Harmful Algal Blooms formation. Life in the Mediterranean Sea: a look at habitat changes.

[b19] Gómez P, Buckling A (2011). Bacteria-phage antagonistic coevolution in soil. Science.

[b20] Guillard R, Stein J (1973). Division rates. Handbook of phycological methods: culture methods and growth measurements.

[b41] Guillard RRL, Hargraves PE (1993). Stichochrysis immobilis is a diatom, not a chrysophyte. Phycologia.

[b21] Hall AR, Scanlan PD, Morgan AD, Buckling A (2011). Host-parasite coevolutionary arms races give way to fluctuating selection. Ecol. Lett.

[b22] Koskella B (2013). Phage-mediated selection on microbiota of a long-lived host. Curr. Biol.

[b23] Koskella B, Lively CM (2009). Frequency-dependent selection during experimental coevolution of a freshwater snail and a sterilizing trematode. Evolution.

[b24] Kover PX, Fox CW, Wolf JB (2006). Evolutionary genetics of host-parasite interactions. Evolutionary genetics: concepts and case studies.

[b25] Lambrechts L, Halbert J, Durand P, Gouagna LC, Koella JC (2005). Host genotype by parasite genotype interactions underlying the resistance of anopheline mosquitoes to Plasmodium falciparum. Malar. J.

[b42] Littell RC, Milliken GA, Stroup WW, Wolfinger RD, Schabenberger O (2006). SAS for mixed models.

[b26] Llaveria G, Garcés E, Ross ON, Figueroa R, Sampedro N, Berdalet E (2010). Small-scale turbulence can reduce parasite infectivity to dinoflagellates. Mar. Ecol. Prog. Ser.

[b27] Luijckx P, Fienberg H, Duneau D (2013). A matching-allele model explains host resistance to parasites. Curr. Biol.

[b28] McKean KA, Yourth CP, Lazzaro BP, Clark AG (2008). The evolutionary costs of immunological maintenance and deployment. BMC Evol. Biol.

[b29] Morran LT, Schmidt OG, Gelarden IA, Parrish RC, Lively CM (2011). Running with the Red Queen: host-parasite coevolution selects for biparental sex. Science.

[b30] Parker MA (1994). Pathogens and sex in plants. Evol. Ecol.

[b40] Quinn G, Keough MJ (2002). Experimental design and data analysis for biologists.

[b31] Rauch G, Kalbe M, Reusch TBH (2006). One day is enough: rapid and specific host-parasite interactions in a stickleback-trematode system. Biol. Lett.

[b32] Richlen ML, Erdner DL, McCauley LAR, Libera K, Anderson DM (2012). Extensive genetic diversity and rapid population differentiation during blooms of *Alexandrium fundyense* (Dinophyceae) in an isolated salt pond on Cape Cod, MA, USA. Ecol. Evol.

[b33] Salvaudon L, Héraudet V, A Shykoff J (2007). Genotype-specific interactions and the trade-off between host and parasite fitness. BMC Evol. Biol.

[b34] Schulenburg H, Ewbank JJ (2004). Diversity and specificity in the interaction between *Caenorhabditis elegans* and the pathogen *Serratia marcescens*. BMC Evol. Biol.

[b35] Taylor FJR (1968). Parasitism of the toxin-producing dinoflagellate *Gonyaulax catanella* by the endoparasitic dinoflagellate *Amoebophrya ceratii*. J. Fish. Res. Board Can.

[b36] Thrall PH, Laine A-L, Ravensdale M, Nemri A, Dodds PN, Barrett LG (2012). Rapid genetic change underpins antagonistic coevolution in a natural host-pathogen metapopulation. Ecol. Lett.

[b37] Toth GB, Norén F, Selander E, Pavia H (2004). Marine dinoflagellates show induced life-history shifts to escape parasite infection in response to water-borne signals. Proc. R. Soc. B.

[b38] Woolhouse MEJ, Webster JP, Domingo E, Charlesworth B, Levin BR (2002). Biological and biomedical implications of the co-evolution of pathogens and their hosts. Nat. Genet.

