# Supplementary material for: The potential for arms race and Red Queen coevolution in a protist host–parasite system
Source: Ecol Evol. 2014 Dec 2;4(24):4775–85. doi: 10.1002/ece3.1314 (PMC4278826; doi:10.1002/ece3.1314)
Supplement: Supplementary file 1 [file ece30004-4775-sd1.docx]

Tab S1. Details of host clones.

| Clonal strain key name* | Strain name | Locality of isolation | Year of isolation |
| --- | --- | --- | --- |
| H1 | AMP13 | Palma de Mallorca (Med, Spain) | 1995 |
| H2 | VGO942 | Adriatic Sea (Italy) | 2008 |
| H3 | VGO874 | Boughrara (Tunis) | 2006 |
| H4 | Min3 | Arenys (Med, Spain) | 2002 |
| H5 | AL10 | Estartit (Med, Spain) | 2002 |
| H7 | VGO577 | Girona (Med, Spain) | 2002 |
| H8 | VGO707 | Delta de l´Ebre (Med, Spain) | 2003 |
| H9 | VGO722 | Cambrils (Med, Spain) | 2003 |
| H10 | VGO663 | Sardinia | 2003 |

*A clone H6 was included initially, but this isolate did not grow well during the initial cloning, and so was excluded from the experiment at an early stage (before inoculations).

Tab S2. Details of parasite clones.

| Clonal strain name | Locality of isolation (Mediterranean sea, Spain) | Date of isolation |
| --- | --- | --- |
| P1 | Estartit | 21/05/2011 |
| P2 | Arenys | 15/02/2012 |
| P3 | Olímpic | 03/06/2011 |
| P4 | Vilanova | 07/05/2011 |
| P5 | Estartit | 17/06/2011 |
| P6 | Tarragona | 20/06/2011 |
| P7 | Vilanova | 2009 |
| P8 | Cambrils | 06/06/2011 |
| P9 | Vilanova | 07/05/2011 |
| P10 | Vilanova | 23/06/2011 |
